# The relationship between duration and quality of sleep and upper respiratory tract infections: a systematic review

**DOI:** 10.1093/fampra/cmab033

**Published:** 2021-05-17

**Authors:** Charlotte H Robinson, Charlotte Albury, David McCartney, Benjamin Fletcher, Nia Roberts, Imogen Jury, Joseph Lee

**Affiliations:** 1 Department of Medical Sciences, University of Oxford, Oxford, UK; 2 Nuffield Department of Primary Care Health Sciences, University of Oxford, Oxford, UK; 3 Bodleian Health Care Libraries, University of Oxford, Oxford, UK

**Keywords:** Prevention, sleep duration, sleep quality, systematic review, upper respiratory tract infection, URTI

## Abstract

**Background:**

Upper respiratory tract infections (URTIs) are common, mostly self-limiting, but result in inappropriate antibiotic prescriptions. Poor sleep is cited as a factor predisposing to URTIs, but the evidence is unclear.

**Objective:**

To systematically review whether sleep duration and quality influence the frequency and duration of URTIs.

**Methods:**

Three databases and bibliographies of included papers were searched for studies assessing associations between sleep duration or quality and URTIs. We performed dual title and abstract selection, discussed full-text exclusion decisions and completed 50% of data extraction in duplicate. The Newcastle–Ottawa Quality Assessment Scale assessed study quality and we estimated odds ratios (ORs) using random effects meta-analysis.

**Results:**

Searches identified 5146 papers. Eleven met inclusion criteria, with nine included in meta-analyses: four good, two fair and five poor for risk of bias. Compared to study defined ‘normal’ sleep duration, shorter sleep was associated with increased URTIs (OR: 1.30, 95% confidence interval [CI]: 1.19–1.42, *I*^2^: 11%, *P* < 0.001) and longer sleep was not significantly associated (OR: 1.11 95% CI: 0.99–1.23, *I*^2^: 0%, *P* = 0.070). Sensitivity analyses using a 7- to 9-hour baseline found that sleeping shorter than 7–9 hours was associated with increased URTIs (OR: 1.31, 95% CI: 1.22–1.41, *I*^2^: 0%, *P* < 0.001). Sleeping longer than 7–9 hours was non-significantly associated with increased URTIs (OR: 1.15, 95% CI: 1.00–1.33, *I*^2^: 0%, *P* = 0.050, respectively). We were unable to pool sleep quality studies. No studies reported on sleep duration and URTI severity or duration.

**Conclusions:**

Reduced sleep, particularly shorter than 7–9 hours, is associated with increased URTIs. Strategies improving sleep should be explored to prevent URTIs.

## Lay Summary

It is widely believed that poor sleep increases people’s chances of catching coughs, colds and other upper airway infections. UK government advice states that poor sleep and catching a cold or the flu could be related and suggests most individuals need 8 hours sleep a night. Studies have helped to explain the link between sleep and infections by showing that shortened sleep reduces the body’s ability to fight infections. Studies in humans that look at the link between sleep and catching a cold or other airway infection have mostly been small and have conclusions that differ. We set out to investigate whether the quality of sleep (how ‘well’ you sleep) and the quantity of sleep (how ‘long’ you sleep) influence a person’s likelihood of getting an upper airway infection. We found that shorter sleep than normal resulted in increased chances of having an upper airway infection, whereas longer sleep did not. We also found that sleeping for shorter or longer than 7–9 hours per night increased the likelihood of having an upper airway infection. Our results are important for informing conversations between patients and doctors around sleep and for encouraging the investigation of the impact of sleep on more serious infections.

**Key Messages**

- This is the first systematic review of sleep duration and quality on upper respiratory tract infections (URTIs).
- We included nine studies in meta-analyses out of 5146 titles.
- Sleeping less than study defined ‘normal’ is associated with increased URTIs.
- Sleeping for less than 7–9 hours is associated with increased URTIs.
- Sleeping for more than 7–9 hours is associated with increased URTIs.
- Data from studies on sleep quality and URTIs are lacking.

## Introduction

Upper respiratory tract infections (URTIs) are typically viral infections (1) of the URT, including the nose, sinuses, pharynx and larynx. Adults experience ~two to three URTIs per year (2), and with the total direct and indirect cost of URTIs on the UK economy surpassing £76 million (3), URTIs place a large burden on the economy and medical services. Due to the issue of growing antibiotic resistance, in 2019 the UK government set a goal to reduce UK antibiotic use in humans by 15% by 2024 (4). A recent study assessed the contribution of URTIs to primary care antibiotic prescribing rates in England, using data recorded between 2013 and 2015 from UK primary care records. It was found that of the 69% of antibiotic prescriptions linked to a clinical condition or body system, 10.4% were for cough symptoms, 7.68% for a sore throat and 6.67% for URTIs (5). URTIs are mostly self-limiting, but are significant because they are common, and for their impact on antibiotic prescribing.

‘Poor sleep’ is commonly believed to increase susceptibility to infection. UK government advice states poor sleep and catching a cold or the flu could be related and recommends most individuals need 8 hours sleep a night (6). The National Sleep Foundation suggests normal sleep for 18- to 64-year olds is 7–9 hours, but acknowledges 6–11 hours may be appropriate for some 18- to 25-year olds, and 6–10 hours for some 26- to 64-year olds (7). The impact of sleep on immunity has been systematically reviewed: studying sleep in laboratory settings showed sleep deprivation produces a diminished cytokine response to lipopolysaccharide, a component of gram-negative bacteria (8). One study found that sleep deprivation reduces the efficacy of the hepatitis A vaccine (9).

Studies on sleep and URTI occurrence have conflicting results. Viral challenge studies showed that short sleep and sleep disturbance are associated with increased URTIs (10–12), yet a study in Sweden found that sleep duration and quality were not associated with increased URTIs (13). We therefore aimed to bring together the entirety of the clinical evidence in the first systematic review of sleep and URTIs.

## Methods

### Registration

A protocol was prospectively registered in PROSPERO (CRD42018097466).

### Outcomes

The primary outcome was symptomatic URTI, expressed as the rate of occurrence of URTI in participants, or the proportion of patients who experienced ≥1 URTIs. The secondary outcomes were the severity and duration of the URTIs, and all clinically relevant outcomes reported.

### Exposures

We compared the study defined ‘normal sleep’ duration with longer or shorter durations. ‘Short’ sleep was defined as sleep durations lower than study defined ‘normal’, and ‘long’ sleep was defined as sleep durations higher than study defined ‘normal’. To overcome study variability in defining ‘normal sleep duration’, we pre-planned two sensitivity analyses using 7–8 and 7–9 hours of sleep as the reference group, based on the National Sleep Foundation’s (7) definition of ‘normal sleep’ for adults aged ≥65 years and 18–65 years, respectively.

### Eligibility criteria

Studies were eligible for inclusion if they examined the association between sleep quality or duration and URTIs. We included adults, aged ≥18 or as the study defined, of any sex, in any setting. We included studies that diagnosed URTIs via clinician assessment, laboratory techniques or self-report. We included studies measuring sleep subjectively or objectively. There were no restrictions on language or year of publication. For interventional studies involving infection with viruses, a minimum follow-up period of 5 days was pre-specified, as the common cold incubation period is 12 hours to 5 days (14). Exclusion criteria: (i) studies looking solely at populations with sleep or chronic disorders, (ii) studies looking solely at children, (iii) studies where sleep duration, sleep quality and number of URTIs were measured but an effect could not be calculated, (iv) patient follow-up rate below 80%, (v) protocol-only publications or (vi) case series and case reports.

### Information sources and search strategy

Initially databases were searched from their inception up to 31 May 2018: EMBASE(OvidSP) [1974–present], MEDLINE(OvidSP) [1946–present] and PsycINFO(OvidSP) [1806–present]. Reference lists of included articles were reviewed for extra citations. The search strategy (Supplementary Table S1) was developed in consultation with an information specialist (NR). Search terms were reviewed by a sleep researcher, Nick Meyer, at King’s College London; the URTI terms were developed from those used in a study by Merlin Wilcox, Clinical Lecturer at the University of Southampton, and reviewed by Oliver van Hecke, Clinical Lecturer at the University of Oxford; and the patient and public involvement group for the Nuffield Department of Primary Care Health Sciences, to ensure all possible search terms were included. The database searches were updated to include any studies published between 1 January 2018 and 10 January 2020.

### Study selection, data collection and quality assessment

Titles and abstracts from the first database searches on 31 May 2018 were screened for eligibility by two reviewers (CR and IJ) independently using Rayaan software (15). Two reviewers (CR and JL) double screened the studies’ titles and abstracts from the database searches that ran on 10 January 2020. Discrepancies were resolved by discussion. Duplicates were removed (CR). Studies were imported into Mendeley version 1.19.3, a reference manager. CR led a full-text review and discussed exclusion decisions with the study team (CA and JL). CR extracted relevant data. The first five papers were extracted and quality assessed in duplicate (CR and IJ) for accuracy. Disagreements were resolved by discussion. The Newcastle–Ottawa Quality Assessment Scale (NOS) (16) was used to assess study bias of cross-sectional and cohort studies. The results from the NOS were converted into the Agency for Healthcare Research and Quality standards of ‘good’, ‘fair’ and ‘poor’ quality. As no explicit conversion guidance exists, conversion thresholds from a prior publication were used (17).

### Statistical analysis and reporting

Data analysis followed the Cochrane Handbook for systematic reviews of interventions (18). Random effects meta-analysis was performed where possible; if not, a narrative synthesis of included studies was performed. We estimated pooled odds ratios (ORs) and 95% confidence intervals (CIs) for URTI occurrence in each sleep duration group. The *I*^2^ statistic assessed statistical heterogeneity, which was explored with sensitivity analyses where appropriate. Funnel plots and Egger’s tests, if appropriate, were planned to assess publication bias if appropriate. We conducted sensitivity analyses using 7–8 and 7–9 hours of sleep per night as the reference group. We assessed the impact of studies with a high risk of bias in sensitivity analyses excluding their data. Analysis was conducted using Review Manager 5.3 software (19). We used the Preferred Reporting Items for Systematic Review and Meta-Analysis (PRISMA) checklist (20).

## Results

### Study selection and characteristics

**Figure 1.**
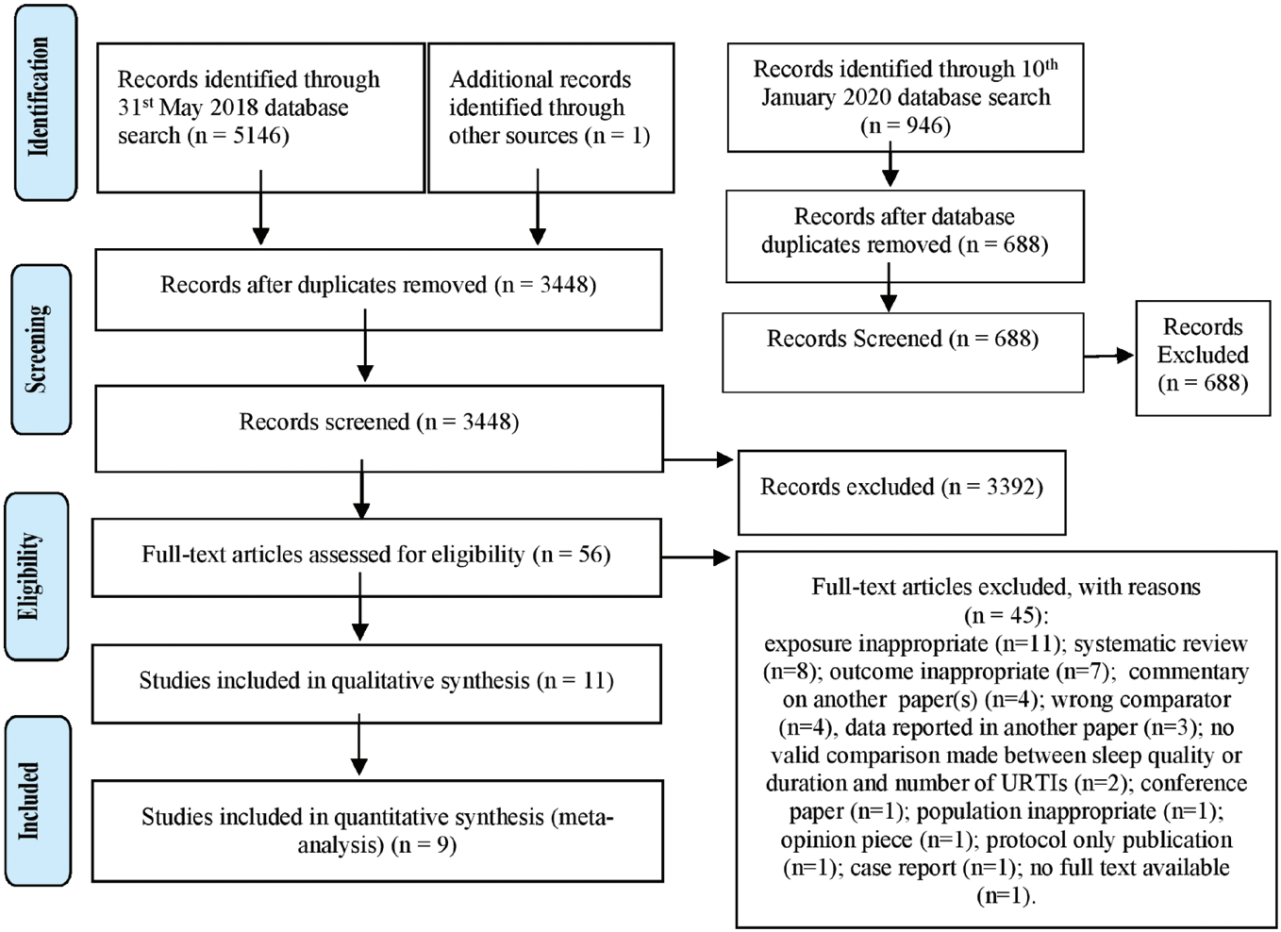
PRISMA 2009 flow diagram for identification, screening, eligibility and inclusion of studies (2019–20). PRISMA, Preferred Reporting Items for Systematic Reviews and Meta-Analyses; URTIs, upper respiratory tract infections.

The searches ran on 31 May 2018 found 5146 studies (Fig. 1). We removed 1699 duplicates. Citation searching gave one extra study. Title and abstract screening removed 3392 studies, full-text review excluded 45 studies, resulting in 11 eligible studies (10–13,21–27), with nine included in the meta-analysis (10–13,23–27). Two eligible studies (21,22) were not included in the meta-analysis, as they did not report sufficient quantitative information. One study (15) included in the meta-analysis presented pooled data from three similar studies from the same research group: Prather 2017a was referred to as ‘The Pittsburgh Cold Study 2’ (PCS2), Prather 2017b as ‘The Pittsburgh Cold Study 3’ (PCS3) and Prather 2017c as ‘The Pittsburgh Mind-Body Center Study’ (PMBC) in the Prather 2017 paper (11). Prather 2015 (10) and Cohen 2009 (12) report on the same participants as in Prather 2017b and Prather 2017c, respectively. They were included in this review as they presented additional data that was not reported in the Prather 2017 data obtained from the authors. Statistical analyses include data from Prather 2017 or one or both of Prather 2015 and Cohen 2009, to prevent double inclusion of participants. Table 1 presents the characteristics of the included studies in the qualitative synthesis. The papers present results from 66 229 patients, across five countries. Three cross-sectional studies (23–25) and six cohort studies (10–13,26,27) were included in the meta-analysis. There were no randomized controlled trials. The database searches to 10 January 2020 found 946 studies. Two hundred fifty-eight duplicates were removed. Title and abstract screening removed 688 studies and no additional studies were included in the review from this update.

**Table 1.** Characteristics of included studies in the systematic review and meta-analysis (2019–20)

Study (year, reference number)	Design	Number of participants	Age information (years)	Gender	Country	Reference sleep duration category	Outcome ascertainment	Sleep duration ascertainment	Length of assessment of outcome	Study setting
d’Arcy (2000, 24)	Cross-sectional	185	≥18	F	USA	≥8 hours	Self-report	Subjective	2 weeks	Retrospective phone interview
Prather (2015, 10)	Cohort	164	18–55	94M, 70F	USA	>7 hours	Lab diagnosed	Objective	5 days	Quarantine
Prather (2017, 11)	Cohort	734 (Prather 2017a: 331, Prather 2017b: 212, Prather 2017c: 191^a^)	Data from authors did not give demographic details	Data from authors did not give demographic details	USA	≥8 hours	Lab diagnosed	Subjective	5 days for rhinovirus, 6 days for influenza	Quarantine
Ghilotti (2018, 13)	Cohort	1635	25–64	NR	Sweden	6–7 hours	Self-report	Subjective	9 months	One-off questionnaire on sleep and self- reporting of naturally acquired illness
Chan (2018, 26)	Cohort	160	20–70	38M 122F	Taiwan	NA^c^	Self-report	Subjective	Varied per participant and not reported but all >2 months	Web diaries
Shibata (2018, 25)	Cross-sectional	39 524	40–79	26 975M 12 549F	Japan	7 hours	Self-report	Subjective	NA	One-off questionnaire
Cohen (2009, 12)	Cohort	153	21–55	78M 75F	USA	≥8 hours	Lab diagnosed	Subjective	5 days	Quarantine
Wentz (2018, 27)	Cohort	651	18–25	436M 215F	England	7–9 hours	Self-report	Objective	13 weeks	Military training programme
Prather (2016, 23)	Cross-section-al	22 726	Mean 46.2	M and F	USA	7–8 hours	Self-report^d^	Subjective	30 days	Retrospective questionnaire
Albright (2011, 21)	Cross-section-al	21	Under-graduated students	NR	USA	NR	Lab diagnosed	Subjective	NA	University practical class
Cohen (1997, 22)	Cohort	276	18–55	125M 151F	USA	NA^b^	Lab diagnosed	Subjective	5 days	Quarantine

Characteristics of included studies in the systematic review and meta-analysis. M, male; F, female; NR, not reported; NA, not applicable.

^a^Study presented pooled data from three similar studies from the same research group; Prather 2017a is referred to as PCS2 in Prather 2017, Prather 2017b as PCS3 and Prather 2017c as PBMC. It is of note that Prather 2015 reports on the same participants as in Prather 2017b, Prather 2015 is included in this review as it presented additional data that was not included in the Prather 2017 data set obtained from the authors. Cohen 2009 reports on the same participants as in Prather 2017c, Cohen 2009 is included in this review as it presented additional data that was not included in the Prather 2017 data set obtained from the authors.

^b^Study looked only at sleep quality, did not assess sleep duration.

^c^Study reported sleep duration as a continuous variable and did not report a reference group.

^d^The study presented data on respiratory tract infections including head and chest colds, influenza, pneumonia and ear infections. Our review only included the head and chest colds data to ensure we met our URTI inclusion criteria.

### Risk of bias within studies

Tables 2 and 3 present the risk of bias assessment for included cross-sectional and cohort studies. No study received the highest possible mark of all nine stars: each was judged to have a risk of bias or lack of clarity in methodological reporting. Results of the NOS were converted to AHRQ standards: four studies were good (10–12,22), two were fair (23,25) and five were poor (13,21,24,26,27). Only one study (23) was truly representative of the general population; the other studies looked at select groups such as military recruits, mothers of young children, university students or volunteers responding to advertisements. All studies addressed confounders, with seven studies (10–13,22,25,27) addressing what we considered most important: being chronically ill or immunocompromised.

**Table 2.** Newcastle–Ottawa risk of bias assessment scale results for cross-sectional studies included in the qualitative synthesis (2019–20)

Study (year)	Selection					Comparability of cohorts^a^	Outcome			Total score	AHQR standard (good/fair/poor)^b^
	Representativeness of exposed cohort	Sample size	Non-respondents	Ascertainment of the exposure	Total		Assessment of outcome	Statistical test	Total		
d’Arcy (2000)	–	–	*	*	2	1	*	–	1	4	poor
Shibata (2018)	–	–	*	*	2	2	*	*	2	6	fair
Prather (2016)	*	–	–	*	2	1	*	*	2	5	fair
Albright (2011)	–	–	–	*	1	1	*	–	1	3	poor

Newcastle–Ottawa risk of bias assessment scale results for cross-sectional studies included in the qualitative synthesis. The results were converted in AHQR standards of ‘good’, ‘fair’ or ‘poor’. Each category within selection and outcome can be awarded up to one star, comparability can be awarded up to two stars.

^a^Two stars can be awarded for comparability. First star is awarded if the study controls for chronic illness or being immunocompromised. Second star is awarded if the study controls for any other factor.

^b^Thresholds for converting the Newcastle–Ottawa scales to AHRQ standards (good, fair and poor): Good quality: three or four stars in selection domain AND one or two stars in comparability domain AND two or three stars in outcome/exposure domain. Fair quality: two stars in selection domain AND one or two stars in comparability domain AND two or three stars in outcome/exposure domain. Poor quality: zero or one star in selection domain OR zero stars in comparability domain OR zero or one star in outcome/exposure domain.

**Table 3.** Newcastle–Ottawa risk of bias assessment scale results for cohort studies included in the qualitative synthesis (2019–20)

Study (year)	Selection					Comparability of cohorts^a^	Outcome				Total Score	AHQR standard (good/fair/ poor)^b^
	Representativeness of exposed cohort	Selection of non-exposed cohort	Ascertainment of exposure	Demonstration outcome of interest not present at the start of the study	Total		Assessment of outcome	Follow-up long enough for outcomes to occur?	Adequacy of follow-up of cohorts	Total		
Prather (2015)	**–**	*	*	*	3	2	*	*	–	2	7	Good
Prather (2017)	**–**	*	*	*	3	2	*	*	*	3	8	Good
Ghilotti (2018)	**–**	*	–	*	2	2	–	*	–	1	5	Poor
Chan (2018)	**–**	*	–	*	2	1	–	–	–	0	3	Poor
Cohen (2009)	**–**	*	*	*	3	2	*	*	*	3	8	Good
Wentz (2018)	**–**	*	–	–	1	2	*	*	*	3	6	Poor
Cohen (1997)	**–**	*	*	*	3	2	*	*	–	2	7	Good

Newcastle**–**Ottawa risk of bias assessment scale results for cohort studies included in the qualitative synthesis. The results were converted in AHQR standards of ‘good’, ‘fair’ or ‘poor’. Each category within selection and outcome can be awarded up to one star, comparability can be awarded up to two stars.

^a^Two stars can be awarded for comparability. First star is awarded if the study controls for chronic illness or being immunocompromised. Second star is awarded if the study controls for any other factor.

^b^Thresholds for converting the Newcastle**–**Ottawa scales to AHRQ standards (good, fair and poor): Good quality: three or four stars in selection domain AND one or two stars in comparability domain AND two or three stars in outcome/exposure domain. Fair quality: two stars in selection domain AND one or two stars in comparability domain AND two or three stars in outcome/exposure domain. Poor quality: zero or one star in selection domain OR zero stars in comparability domain OR zero or one star in outcome/exposure domain.

### Short sleep and URTI occurrence

**Figure 2.**
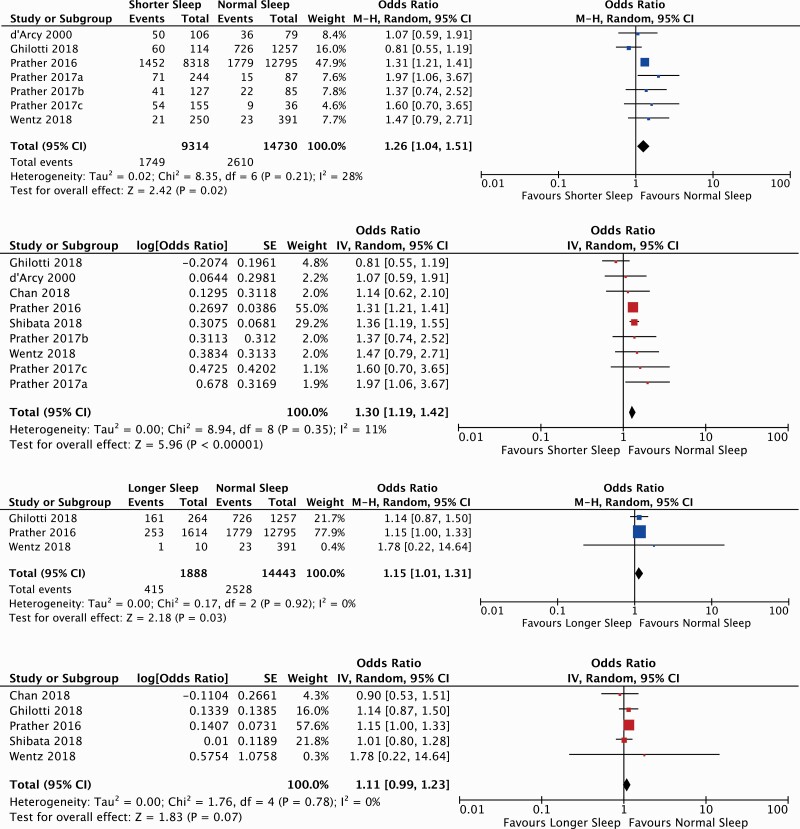
Forest plots for random effects meta-analyses using ‘normal sleep’ as reference and ‘short sleep’ or ‘long sleep’ as comparator (2019–20). Pooled results compare the number of individuals who experienced ≥1 URTIs with sleep duration. (**a**) Forest plot comparing ‘Short sleep vs normal sleep’ for UTRI occurrence. Calculated using the number of people and URTI event. Results are expressed as odds ratios (ORs) and 95% confidence intervals (95% CIs). Pooled analysis: OR: 1.26, 95% CI: 1.04–1.51, *I*^2^: 28%, *P* = 0.02 (2019–20). (**b**) Forest plot for ‘Short sleep vs normal sleep’ for URTI occurrence. Calculated using ORs and CIs from adjusted regression models. Results are expressed as ORs and 95% CIs. Pooled analysis: OR: 1.30, 95% CI: 1.19–1.42, *I*^2^: 11%, *P* < 0.001 (2019–20). (**c**) Forest plot comparing ‘Long sleep vs normal sleep’ for URTI occurrence. Calculated using the number of people and URTI events. Results are expressed as ORs and 95% CIs. Pooled analysis: OR: 1.15, 95% CI: 1.01–1.31, *I*^2^: 0%, *P* = 0.03 (2019–20). (**d**) Forest plot comparing ‘Long sleep vs normal sleep’ for UTRI occurrence. Calculated using ORs and CIs from adjusted regression models. Results are expressed as ORs and 95% CIs. Pooled analysis: OR: 1.11, 95% CI: 0.99–1.23, *I*^2^: 0%, *P* = 0.07 (2019–20).

We conducted two meta-analyses to investigate the relationship between short sleep and URTI occurrence: one used number of people and URTI events in each sleep group (OR: 1.26, 95% CI: 1.04–1.51, *I*^2^: 28%, *P* = 0.020, seven studies (10,11,13,23,24,27), 24 044 individuals, Fig. 2a), the other used ORs and CIs from adjusted regression models to calculate URTI presence (OR: 1.30, 95% CI: 1.19–1.42, *I*^2^: 11%, *P* < 0.001, nine studies (10–13,23–27), Fig. 2b).

### Longer sleep and URTI occurrence

We conducted two meta-analyses to investigate the relationship between longer sleep (compared to normal) and URTI occurrence: one used number of people and URTI events in each sleep group (OR: 1.15, 95% CI: 1.01–1.31, *I*^2^: 0%, *P* = 0.030, three studies (13,23,27), 16 331 individuals, Fig. 2c), the other used ORs and CIs for URTI presence (OR: 1.11 95% CI: 0.99–1.23, *I*^2^: 0%, *P* = 0.070, five studies (13,23,25–27), Fig. 2d).

### Sleep quality

Eight studies (10–13,22–24,26) measured sleep quality, but data was not used in the meta-analysis because measures were too heterogeneous. Four studies (12,22–24) reported associations between poor sleep quality and URTIs and four (10,11,13,26) found no association. Sleep quality was measured in 11 different ways (Supplementary Table S2). Self-reported sleep efficiency (the proportion of time in bed spent sleeping described as a percentage) was measured in four studies (10–12,22). Two studies found associations (12,22) between lower self-reported sleep efficiency and increased risk of developing colds, and two found no associations (10,11). Meta-analysis was not possible to investigate sleep efficiency and URTI occurrence, as the sleep efficiency groups across studies were too heterogeneous to be pooled. ‘Abnormal’ sleep efficiency was the only sleep efficiency group reported in more than one study. Data from the two studies (12,22) reporting ‘abnormal’ sleep efficiency could not be combined as we considered these individuals likely had disorders beyond sleep efficiency, as their reported sleep efficiencies were much lower than the reported mean, and so were outside the realms of ‘normality’. Subjective sleep quality was measured in three studies: one measured it on a 0–3 scale (11), one study (13) measured it as one of two categories (quite good/good, or neither bad nor good/quite bad/bad) and one study (22) did not report its measurement. None of the studies found an association between subjective sleep quality and cold/URTI risk. The additional sleep quality measures reported in papers were unique to their study. One study (10) measured sleep quality objectively using wrist actigraphy and did not show an association between sleep quality and URTIs.

### Secondary outcomes

The severity or duration of URTIs was measured in three studies (21,24,27), but sufficient information was not reported to calculate their association with sleep. No other clinically relevant outcomes were reported.

### Risk of bias across studies

None of the meta-analyses included 10 or more studies; therefore, we could not reliably assess publication bias using funnel plots or Egger’s tests (18).

### Additional analysis

As pre-specified, to explore the issue of ‘normal sleep duration’ being reported differently across studies, we performed sensitivity analyses using 7–8 and 7–9 hours sleep as the reference groups and comparing the reference with longer or shorter sleep. Three studies were included in the sensitivity analyses using 7–8 hours of sleep as a reference (12,24,25). There was a non-significant trend between shorter than 7–8 hours sleep and increased URTIs: OR: 1.13, 95% CI: 0.99–1.29, *I*^2^: 0%, *P* = 0.060 (Supplementary Figure S1). Longer than 7–8 hours sleep was not associated with increased URTIs: OR: 0.94, 95% CI: 0.76–1.16, *I*^2^: 0%, *P* = 0.530 (Supplementary Figure S2). Two sensitivity analyses used a 7- to 9-hour reference group: one with shorter sleep the comparator (included two studies (24,27) with 21 754 people), the other using longer sleep (included two studies (23,27) with 14 810 people). There was a significant association between shorter than 7–9 hours and increased URTIs: OR: 1.31, 95% CI: 1.22–1.41, *I*^2^: 0%, *P* < 0.001 (Supplementary Figure S3). Longer than 7–9 hours sleep and increased URTIs had a non-significant association: OR: 1.15, 95% CI: 1.00–1.33, *I*^2^: 0%, *P* = 0.050 (Supplementary Figure S4).

We performed two pre-specified sensitivity analyses removing four studies assessed as ‘poor’ for risk of bias from the meta-analyses calculated using OR and CIs. Removing these studies (13,24,26,27) from the meta-analysis with ‘shorter sleep’ as the comparator caused *I*^2^ to drop from 11% to zero and changed the OR from 1.30 (1.19–1.42, *P* < 0.00001) to 1.33 (1.25–1.42, *P* < 0.00001). Removing poor quality studies (13,26,27) from the meta-analysis with ‘longer sleep’ as the comparator, kept *I*^2^ at 0% and changed the estimates from 1.11 (0.99–1.16, *P* = 0.07000) to 1.11 (0.98–1.25, *P* = 0.09000).

## Discussion

### Main findings

The study defined ‘short sleep’ was associated with increased URTIs, whereas ‘long sleep’ was not when comparing against the study defined ‘normal sleep’. From the sensitivity analyses using 7- to 8-hour and 7- to 9-hour reference groups, our findings suggest that sleeping for shorter than 7–9 hours per night could increase the occurrence of URTIs. Sleeping longer than 7–9 hours was non-significantly associated with increased URTIs (*P* = 0.050). The 7- to 8-hour and 7- to 9-hour reference group sensitivity analyses were calculated by pooling two and three studies, respectively, with one study in each analysis significantly dominating the weighting. The quality of studies was mixed, with only one study (23) awarded a star for being truly representative of the general population. Additional sensitivity analyses supported conclusions from meta-analyses.

There is little evidence addressing the association between sleep quality and URTI occurrence, and we did not meta-analyse it due to variable measurements of sleep quality. Apart from sleep efficiency, subjective sleep quality was the only other quality assessment measured across studies (11,13,22) and meta-analysis was not possible. No significant association was found between subjective sleep quality and cold/URTI risk, suggesting subjective sleep quality does not influence URTI occurrence. No studies directly investigated the relationship between sleep duration and URTI severity or duration.

### Strengths and limitations

The strengths of this review are that we brought together all published findings in a systematic review, following PRISMA guidelines, and our protocol was published prospectively on PROSPERO. The comprehensive search strategy was developed in consultation with an information specialist and subject experts, so is unlikely to have missed studies that would change the results. To our knowledge, this is the first systematic review looking at the effect of sleep on URTI occurrence and it includes all the available evidence. The included studies present data from 66 229 people across five countries, so results are broadly applicable.

The systematic review was limited by the available evidence. Quality was variable and the small number of included studies meant assessment of publication bias as planned was not possible. The lack of a clearly defined ‘normal’ sleep duration and quality led to variability in measurements reported, limiting meta-analysis. We were able to address this with sensitivity analyses. The association between sleep quality and URTI occurrence could not be quantitatively assessed, as outcome measures were too heterogeneous. Included studies had very limited data on secondary outcomes, so an effect could not be calculated. Many studies included self-diagnoses of URTIs. This is a limitation, but self-diagnosis of colds is usually accurate (28) and false-positive influenza reports are rare (29), so subjective outcome measurement is likely to be adequate. The outcome measured was ≥1 URTIs which is dependent on patient follow-up. All the included cohort studies had a follow-up length of 13 weeks or shorter; with the exception of one study (26) which did not report patient follow-up length and one study (13) with a follow-up length of 9 months; however, neither study was included in the sensitivity analyses with 7–8 hours and 7–9 hours sleep as reference. The different follow-up times could have increased heterogeneity of results, but heterogeneity was low. To check we performed a *post hoc* meta-regression of effect sizes against follow-up time for seven studies examining short sleep (11,13,23,24,27). There was no association between effect size and follow-up. We were unable to perform a similar analysis for studies of long sleep as there were only three studies (13,23,27).

### Comparison with existing literature and implications for practise

This is the first systematic review examining sleep quality and duration and URTI occurrence. It has been previously established that sleep has a regulatory role on the immune system (30,31). Immune parameters in human blood show systematic fluctuations; the influence of sleep on these temporal changes has been separated from those of circadian processes in two studies (30,32). Studies show that sleep deprivation activates the hypothalamus–pituitary–adrenal axis and sympathetic nervous system (33), which results in diminished immune response: reduced T-cell proliferation (34,35), T-helper cell 1 cytokine production (34,36) and natural killer cell cytotoxicity (37). A systematic review and meta-analysis of cohort and experimental sleep deprivation studies found that sleep disturbance and long sleep duration are associated with increased systemic inflammatory markers (interleukin-6 and C-reactive protein) (38). However, whether sleep’s role on the immune system influences URTI occurrence had not been systematically reviewed. Despite these relationships between sleep and markers of the immune system, the mechanism through which sleep duration may influence URTI occurrence is unknown. One study in our systematic review (11) investigated whether nasal inflammation was a plausible pathway through which sleep influences cold occurrence: the data suggested that nasal cytokines and inflammation do not play a significant role. We found less evidence as to whether longer sleep influences infections. One study with over 56 000 people found that self-reported sleep ≥9 hours increased the risk of pneumonia (39), supporting a possible relationship between long sleep and URTI occurrence.

The direct clinical application of our findings is limited, but they can inform discussions about sleep between patients and their primary care clinicians, and may help facilitate discussions around the broader health implications of short sleep. The commonly held belief that short sleep is associated with URTIs is supported by our review, which is in line with the consensus statement from the American Academy of Sleep Medicine and Sleep Research Society that 7–9 hours of sleep is important for other health conditions (40). URTIs may represent a common opportunity for these discussions, particularly as there is evidence that family practitioners may be more likely to include health-promotion messages in a consultation when they have immediate relevance to their presenting complaint (41).

### Implications for future research

Our review has identified gaps in the evidence base and should prompt examination of sleep association and causality in the occurrence of more clinically serious infections. Future research should explore the role of longer and poor quality sleep on respiratory infections and should use objective measures of sleep quality and duration. For example, studies should consider using the Pittsburgh Sleep Quality Index (42), which was used by only two studies in this review (11,12). Randomized trials of sleep-improvement interventions for the prevention of URTIs could support or provide more evidence for a causal link and inform clinical practice. These could address both prevention and treatment of infections. Future studies would also benefit from expanding outcomes measured to include URTI duration and severity.

## Conclusions

Our findings suggest that sleeping for shorter than 7–9 hours per night could increase URTI occurrence. Sleeping longer than 7–9 hours was non-significantly associated (*P* = 0.05). This review will inform discussions with patients in primary care around sleep and should prompt further research on the broader health implications of short sleep, in particular the association in occurrence of more serious infections, such as SARS-CoV-2 or pneumonia.

## Supplementary Material

cmab033_suppl_Supplementary_Figures

cmab033_suppl_Supplementary_Table_S1

cmab033_suppl_Supplementary_Table_S2

cmab033_suppl_Supplementary_Data

## Declaration

Funding: this study is funded by the National Institute for Health Research (NIHR) School for Primary Care Research. The views expressed are those of the authors and not necessarily those of the NIHR or the Department of Health and Social Care.

Financial disclosure: none.

Non-financial disclosure: none.

Ethical approval: no ethical approval was needed for this study.
