# Supplementary material for: The relationship between duration and quality of sleep and upper respiratory tract infections: a systematic review
Source: Fam Pract. 2021 May 17;38(6):802–10. doi: 10.1093/fampra/cmab033 (PMC8656143; doi:10.1093/fampra/cmab033)
Supplement: cmab033_suppl_Supplementary_Table_S1 [file cmab033_suppl_supplementary_table_s1.docx]

| **#** | **Search History** | **Results** |
| --- | --- | --- |
| 1 | exp *sleep/ | 45909 |
| 2 | (sleep* or "time in bed" or "bed time" or oversleep or asleep).ti,ab. | 157097 |
| 3 | 1 or 2 | 162846 |
| 4 | (upper respiratory tract infection* or upper respiratory infection* or URTIs or "common cold" or influenza or flu or laryngitis or nasolaryngitis or naso-laryngitis or rhinolaryngitis or tonsillitis or rhino-laryngitis or pharyngitis or nasopharyngitis or naso-pharyngitis or rhinitis or sinusitis or nasosinusitis or naso-sinusitis or rhinosinusitis or rhino-sinusitis or tracheitis or supraglottitis or epiglottitis or rhinorrh* or rhinopharyngitis or adenoiditis or laryngotrachitis or tonsillopharyngitis or Croup or Coryza* or Tracheobronchitis or Uvulitis).mp. | 202453 |
| 5 | exp *upper respiratory tract infection/ | 269013 |
| 6 | 4 or 5 | 391524 |
| 7 | 3 and 6 | 2095 |
| 8 | (exp animals/ or nonhuman/) not human/ | 4466404 |
| 9 | 7 not 8 | 2033 |
| 10 | conference*.pt. | 0 |
| 11 | 9 not 10 | 2033 |

Table S1. MEDLINE Search Strategies
