# Supplementary material for: The relationship between duration and quality of sleep and upper respiratory tract infections: a systematic review
Source: Fam Pract. 2021 May 17;38(6):802–10. doi: 10.1093/fampra/cmab033 (PMC8656143; doi:10.1093/fampra/cmab033)
Supplement: cmab033_suppl_Supplementary_Table_S2 [file cmab033_suppl_supplementary_table_s2.docx]

Table 4. Sleep quality assessment measures.

| **Paper ID** | **Sleep Quality Measurement Scoring** | | | | | **Outcome** |
| --- | --- | --- | --- | --- | --- | --- |
| D'arcy, 2000 | Feeling rested when waking (﻿never/rarely, sometimes, frequently/always) | | Sleep interrupted by children (yes/no) | | Number of nights children shared their bed (﻿none, 1–3, 4–13, every night) | Not feeling rested when waking was significantly associated with having ≥2 respiratory symptoms. ﻿Women reporting they rarely/never felt rested were 2.65 times more likely to be ill (95% CI: 1.26,5.55), compared to those reporting frequently /always feeling rested. Sleep interrupted by children (yes compared to no- OR:1.60, 95% CI: 0.84,3.06), or the number of nights children shared the bed (every night compared to none- OR: 1.94 95% CI: 0.80,4.70) was non-significantly associated with having ≥2 respiratory symptoms. ﻿ |
| Prather, 2015 | Self-report sleep efficiency (%) | | Sleep fragmentation measured by actigraphy ﻿(% fragmented) | | | Neither are significant predictors of colds. (﻿﻿fragmentation: b = −0.01, SE = 0.02, P = 0.755, efficiency: not reported) |
| Prather, 2017 | Self-reported sleep efficiency (%) | | Subjective sleep quality ﻿(0-very good to 3-very bad) | | | Sleep efficiency (p=0.20) & quality (p= 0.28) not statistically related to cold risk |
| Ghilotti, 2018 | Subjective sleep quality (quite good/good or neither bad nor good/quite bad/bad) | | | | | No statistical difference in mean number of URTI events between sleep quality groups ﻿(IRR 1.16, 95% CI 0.98–1.37) |
| Chan, 2018 | Staying up later than 1AM once in past three days was used as a proxy measure of sleep quality (yes/no) | | | | | Non-significantly associated with an increased risk of reporting ILI symptoms (estimated OR: 1.43, 95% CI 1.11-1.84) |
| Cohen, 2009 | Self-reported percent nights rested (﻿%) | | Self-reported sleep efficiency (low <92%, middle ≥ 92–98%, high >98–100%, abnormal ≤ 85%)  ﻿ | | | Lower sleep efficiency associated with a greater risk of developing a cold. ﻿In models with 16 control variables, sleep ﻿efficiency was an independent predictor (b=−6.93, SE=3.37, P<0.04). Percent nights rested was unrelated to colds (P>0.17) |
| Cohen, 1997 | Subjective sleep quality (not reported) | Subjective sleep latency and disturbance | | Subjective sleep efficiency (% of time in bed sleeping) | | Increased risk for developing colds if sleep efficiency <0.80. No associations between other sleep measures and colds. Subjective sleep latency and disturbance not reported. |
| Prather, 2016 | Ever had a diagnosed sleep disorder (yes/no) | | Disclosed trouble sleeping to a physician (yes/no) | | | Participants answering ‘yes’ ﻿were more likely to report a head or chest cold (sleep disorder: OR 1.30, 95%CI 1.09-1.54; trouble sleeping: OR 1.29, 95%CI, 1.15-1.45) |

Summary of sleep quality assessment measures. Abbreviations: CI, confidence interval; OR, odds ratio; SE, standard error; IRR, incidence rate ratio; URTI, upper respiratory tract infection; ILI, influenza-like illness.
