# Supplementary material for: The relationship between duration and quality of sleep and upper respiratory tract infections: a systematic review
Source: Fam Pract. 2021 May 17;38(6):802–10. doi: 10.1093/fampra/cmab033 (PMC8656143; doi:10.1093/fampra/cmab033)
Supplement: cmab033_suppl_Supplementary_Data [file cmab033_suppl_supplementary_data.docx]

**Supplementary Data**

Online Supplementary Material

Table S1. Medline Search Strategies

Table S2. Sleep Quality Assessment Measures

Figure S1. 7-8 hours reference, ‘shorter than 7-8 hours’ comparator.

Figure S2. 7-8 hours as reference, ‘longer than 7-8 hours’ comparator.

Figure S3. 7-9 hours reference, ‘shorter than 7-9 hours’ comparator.

Figure S4. 7-9 hours reference, ‘longer than 7-9 hours’ comparator.
